# Supplementary material for: Perioperative Care in Colorectal Cancer Surgery before a Structured Implementation Program of the ERAS Protocol in a Regional Network. The Piemonte EASY-NET Project
Source: Healthcare (Basel). 2021 Dec 31;10(1):72. doi: 10.3390/healthcare10010072 (PMC8775376; doi:10.3390/healthcare10010072)
Supplement: Supplementary file 1 [file healthcare-10-00072-s001.zip › healthcare-1463350-supplementary.pdf]

healthcare

MDPI

Supplemental materials –

Table S1. Description of indicators of ERAS items compliance by phase of care.

Indicators	Rationale of the item	Definition of compliance to the specific item
Preoperative		
Anesthesiological visit time	To assure enough time for preoperative optimization	Visit performed at least 14 days before surgery
Counselling	Patients should receive dedicated preoperative counselling routinely	Counselling provided
Nutritional risk assessment	Preoperative routine nutritional assessment offers the opportunity to correct malnutrition and should be offered	Nutritional risk assessed with MUST score or during a nutritional visit
Anemia correction	Screening and treatment of iron deficency anaemia before surgery	Correction of iron deficency anemia for patients with hemoglobin value <=12 g/dl
No mechanical bowel preparation (colon)	Mechanical bowel preparation has no clear clinical advantage in colonic surgery and should not be used routinely.	Avoid mechanical bowel preparation for colonic surgery
No premedication	To avoid use of long-acting sedative medication before surgery	Avoid long-acting sedative medications is required for compliance to the item
Thromboprophylaxis	Prophylaxis for DVT according to local guidelines	Prophylaxis with either heparin or stockings
Antibiotics prophylaxis	Antibiotics prophylaxis according to local guidelines	Antibiotic prophylaxis prolonged for less than 24 hours
No prolonged fasting	Patients should be allowed to eat up until 6 hours before initiation of anaesthesia	Last food intake between 6 and 18 hours before surgery
Carbohydrate loading	Maltodextrins drinks reduce hunger, thirst, anxiety, postoperative resistance to insulin and to help maintain anabolic state	Maltodextrins administerd before surgery
Intaoperative		
Minimally invasive (MIS) surgery	Minimally invasive approach for colorectal surgery has better short term postoperative outcomes and reduced postoperative stress response	Laparotomic approach or conversion from MIS to open surgery is considered as not compliant
No surgical drainage (colon)	Peritoneal drains show no effect on clinical outcome and should not be used routinely	The use of abdominal drain in colonic surgery is assessed as a not compliant
Epidural anaesthesia in laparotomic	The epidural analgesia in laparotomic approach is the best technique for ensuring an opioid sparing analgesia	Epidural analgesia in laparotomic approach
Prevention of hypothermia	Reliable temperature monitoring and methods to actively warm patients should be employed	Both maintenance of normothermia and prewarming are required for compliance to the item
Fluid normovolemia	Perioperative near-zero fluid balance is the target of the gold directed fluid therapy	Total fluid volume <=4ml/Kg/h during surgery
PONV prevention	A multimodal approach to PONV prophylaxis should be considered	PONV prophylaxis provided
Postoperative items and Follow up		

**Indicators**	**Rationale of the item**	**Definition of compliance to the specific item**
Fluid normovolemia	Net ''near-zero'' fluid and electrolyte balance should be maintained	Total fluid volume <= 2ml/Kg/h in postoperative period
Early removal of i.v.	Maintain the hydro-electrolytic balance by favoring oral fluid intake	Removal of i.v. within day 1 after surgery is required for compliance to the item
Early rehydration	Patients should be encouraged to drink when they are awake and free of nausea after the operation	Oral diet restarted on the day of surgery
Early re-feeding	Most patients can and should be offered food from the day of surgery	Re-feeding within day 1 after surgery
No nasogastric tubes (NGT)	Postoperative NGT should not be used routinely	Removal of NGT whitin day 1 after surgery is required for compliance to the item
Early removal of urin catheter	Patients at low risk should have routine removal of catheter on the first day after surgery	Removal whitin day 1 after surgery
Early mobilization - day 1	Prolonged immobilisation is associated with a variety of adverse effects and patients should therefore be mobilised	At least 2 hours of mobilization the day 1 after surgery
Minimized opioid use	Avoid opioids and apply multimodal analgesia in combination with spinal/epidural analgesia or TAP blocks when indicated	Usage of routinary opioids is considered as not compliant
Early follow-up	Patient follow-up is integral to an ERAS programme	Follow up, also by phone, within 3 days after discharge at home

Table S2. Level of adherence (%) to all the ERAS items, by patient demographic and clinical characteristics, by type of centres (No-ERAS and ERAS).

Variables		No ERAS (N=364)					ERAS (N=79)				
		%	Estimate*	95%CI		p value	%	Estimate*	95%CI		p value
Sex	Female	54.8		ref.			83.7		ref.		
	Male	57.0	-0.41	-2.55	1.74	0.711	78.3	-3.25	-8.49	2.00	0.221
Age groups	<65	55.6		ref.			80.2		ref.		
	65-74	54.0	-2.85	-5.79	0.09	0.058	80.4	-0.65	-7.44	6.13	0.849
	>=75	56.8	-2.61	-5.57	0.35	0.084	79.2	1.51	-4.48	7.51	0.616
Cancer site	Colon	56.2		ref.			81.4		ref.		
	Rectum	54.6	-3.04	-5.36	-0.73	0.010	77.3	-8.89	-14.14	-3.64	0.001
Charlson index	=0	54.1		ref.			79.8		ref.		
	>=1	57.2	2.04	-0.45	4.52	0.109	80.2	-0.37	-6.31	5.58	0.903
ASA score	1-2	55.6		ref.			79.3		ref.		
	3-4	55.9	0.77	-1.74	3.27	0.548	80.6	-0.35	-6.86	6.17	0.916

*Estimated by a multilevel regression model adjusted for patient demographic and clinical characteristics and the centres as random effects.
